# Intranasal BMP9 Ameliorates Alzheimer Disease-Like Pathology and Cognitive Deficits in APP/PS1 Transgenic Mice

**DOI:** 10.3389/fnmol.2017.00032

**Published:** 2017-02-08

**Authors:** Zigao Wang, Lu Xiong, Wenbin Wan, Lijie Duan, Xiaojing Bai, Hengbing Zu

**Affiliations:** ^1^Department of Neurology, Jinshan Hospital, Fudan UniversityShanghai, China; ^2^Department of Anesthesiology, Tinglin HospitalShanghai, China; ^3^Department of Neurology, Zhongshan Hospital, Fudan UniversityShanghai, China

**Keywords:** bone morphogenetic protein 9, Alzheimer’s disease, amyloid β, tau, neuroinflammation, low-density lipoprotein receptor-related protein 1, glycogen synthase kinase-3β

## Abstract

Alzheimer’s disease (AD) is the most common type of dementia and has no effective therapies. Previous studies showed that bone morphogenetic protein 9 (BMP9), an important factor in the differentiation and phenotype maintenance of cholinergic neurons, ameliorated the cholinergic defects resulting from amyloid deposition. These findings suggest that BMP9 has potential as a therapeutic agent for AD. However, the effects of BMP9 on cognitive function in AD and its underlying mechanisms remain elusive. In the present study, BMP9 was delivered intranasally to 7-month-old APP/PS1 mice for 4 weeks. Our data showed that intranasal BMP9 administration significantly improved the spatial and associative learning and memory of APP/PS1 mice. We also found that intranasal BMP9 administration significantly reduced the amyloid β (Aβ) plaques overall, inhibited tau hyperphosphorylation, and suppressed neuroinflammation in the transgenic mouse brain. Furthermore, intranasal BMP9 administration significantly promoted the expression of low-density lipoprotein receptor-related protein 1 (LRP1), an important membrane receptor involved in the clearance of amyloid β via the blood-brain barrier (BBB), and elevated the phosphorylation levels of glycogen synthase kinase-3β (Ser9), which is considered the main kinase involved in tau hyperphosphorylation. Our results suggest that BMP9 may be a promising candidate for treating AD by targeting multiple key pathways in the disease pathogenesis.

## Introduction

Alzheimer’s disease (AD), the most common type of dementia among the elderly, is characterized clinically by progressive memory loss and other cognitive dysfunctions and pathologically by extracellular amyloid β (Aβ) plaques, intracellular neurofibrillary tangles (NFTs) composed of hyperphosphorylated tau, together with the loss of neurons and synapses (Citron, 2010). Aβ is generated from the sequential proteolytic cleavage of amyloid precursor protein (APP) through β- and γ-secretase (Hardy and Selkoe, 2002). A great amount of evidence suggests that the extracellular accumulation, aggregation, and deposition of Aβ play pivotal roles in the pathogenesis of AD by triggering subsequent pathological events, such as tau hyperphosphorylation, neuroinflammation, oxidative stress, neuronal loss and synaptic degeneration. These secondary pathological events then accelerate the progression of AD (Hardy and Higgins, 1992; Tanzi and Bertram, 2005; Bettens et al., 2013). Unfortunately, all the drugs targeting Aβ through immunotherapy or secretase inhibitors have failed to improve cognitive function in AD patients (Doody et al., 2014; Salloway et al., 2014; Lovestone et al., 2015). Very recently, tau aggregation inhibitors also showed no substantial effects against memory decline in AD patients (Gauthier et al., 2016). These failed clinical trials suggest that targeting amyloid plaques or tau aggregation alone might be insufficient to halt the progression of dementia in AD. Thus, multi-target drugs may be the alternative treatments for AD.

Bone morphogenetic protein 9 (BMP9), also known as growth differentiation factor 2, belongs to the transforming growth factor beta (TGF-β) superfamily (Brown et al., 2005). BMP9 signal transduction begins when it binds to transmembrane serine/threonine kinase receptors, which induce the phosphorylation of Smad1, Smad5 and Smad8. Then, the phosphorylated Smads form a complex with Smad4, which translocates into the nucleus, binds to Smad-responsive elements, and eventually induces the transcription of target genes (Bandyopadhyay et al., 2013; Zhong and Zou, 2014). BMP9 has been shown to regulate multiple biological functions, including cartilage formation (Hu et al., 2013), angiogenesis (Levet et al., 2013), and glucose and lipid metabolism (Caperuto et al., 2008) via both autocrine and paracrine pathways (Miller et al., 2000). Recently, BMP9 was determined to be highly expressed in the basal forebrain, where cholinergic neurons are mainly located (López-Coviella et al., 2000). Subsequent evidence revealed that BMP9 was critical for the differentiation, maturation, and phenotype maintenance of cholinergic neurons (López-Coviella et al., 2000, 2005; Schnitzler et al., 2010), suggesting that BMP9 may exert beneficial effects in the treatment of diseases associated with the profound degeneration of cholinergic systems, such as AD (Whitehouse et al., 1982; Grothe et al., 2010).

Indeed, the intracerebroventricular infusion of BMP9 has recently been demonstrated to counteract the cholinergic defects and reduce amyloidosis in APP.PS1/CHGFP mice (Burke et al., 2013). However, whether BMP9 can ameliorate the cognitive deficits in AD, as well as the hyperphosphorylation of tau and neuroinflammation, remains elusive. Here, we demonstrate that a 4-week intranasal BMP9 treatment reduced both the amyloid plaque load and Aβ levels, inhibited tau hyperphosphorylation, suppressed neuroinflammation, and finally reversed the cognitive impairments in APPswe/PSEN1dE9 (APP/PS1) mice.

## Materials and Methods

### Animals

APPswe/PSEN1dE9 mice with a C57BL/6 background were obtained from The Jackson Laboratory (strain name, B6C3-Tg (APPswe, PSEN1dE9) 85Dbo/J; stock number 004462) (Jankowsky et al., 2004). Heterozygous males were bred with wild-type (WT) C57BL/6 females purchased from the Shanghai Research Center for Model Organisms (China). Offspring were genotyped using PCR with primers specific for the APP sequence (Forward: GAATTCCGACATGACTCAGG; Reverse: GTTCTGCTGCATCTTGGACA). Mice not expressing the transgene were adopted as the littermate WT controls. Seven-month-old males were used in all the studies. The animals were housed in groups with free access to water and chow in a temperature-controlled (20–22°C) vivarium and were maintained on a 12-h light/dark cycle. This study and all mouse care and experimental procedures were approved by the Medical Experimental Animal Administrative Committee of Fudan University.

### BMP9 Administration

The intranasal route allows small molecules to rapidly enter the cerebrospinal fluid, followed by subsequent distribution to the brain and spinal cord (Scafidi et al., 2014). Recombinant human BMP9 (R&D Biosciences) was prepared at a concentration of 20 μg/mL using sterile 4 mM HCl containing 0.2% bovine serum albumin (BSA). Each mouse was held ventral side up, and a small, modified 27-French catheter was inserted into either naris. Then, no more than 4 μL of BMP9 was slowly administered, for a total of 50 ng/(g.d). The mouse was held for 1–2 min to ensure absorption. BMP9 was administered daily for 30 days. Mice in the control group were administered an equal amount of vehicle (4 mM HCl containing 0.2% BSA; *n* = 12 per group).

### Morris Water Maze

The Morris water maze test was performed as previously described (McClean et al., 2011). Briefly, the acquisition training paradigm for the Morris water maze consisted of four trials (60-s maximum; 30-min intervals) performed each day for five consecutive days. Escape latency, path length and velocity were recorded during the training days. The probe test was performed 24 h after the last acquisition trial. The platform was removed, and the mice were introduced into the water from a novel entry point. The mice were allowed to swim freely for 1 min while the number of platform crossings and the time spent in the target quadrant were recorded (*n* = 12 per group).

### Contextual Fear Conditioning

The contextual fear conditioning test was performed following previously described methods (Li et al., 2014). In brief, on the day of training, animals were allowed to explore a conditioning chamber for 120 s before the delivery of a 30-s acoustic conditioned stimulus (CS; white noise, 80 dB, 5000 Hz). During the last 2 s of the CS, a shock unconditioned stimulus (US; 0.6 mA) was applied to the grid floor. A total of 3 CS–US pairs with 30-s intervals were presented to each animal. The fear learning test was performed 24 h after training. To evaluate contextual fear learning, animals were returned to the training chamber, and freezing behavior was scored for 3 min. Cued fear learning was assessed 4 h after contextual testing by placing animals in a novel environment, i.e., one with a novel odor, lighting, cage floor and visual cues. After 120 s of free exploration, the animals were exposed to the exact same three CS tones with 30-s intervals without the shock. Freezing responses were recorded. Data are represented as percentages of freezing behavior (*n* = 12 per group).

### Tissue Preparation for Immunofluorescence

Mice were deeply anesthetized with pentobarbital sodium (50 mg/kg, intraperitoneally [i.p.]), and then transcardially perfused with saline and a 4% (w/v) paraformaldehyde fixative in phosphate-buffered saline (PBS, pH 7.4). Whole brains were removed and post-fixed overnight at 4°C, and then immersed consecutively in 20% and 30% sucrose solutions before being embedded in O.T.C. Coronal brain sections (30 μm) were prepared using a Vibratome (Leica). The sections were stored at 4°C in a cryoprotectant (30% glycerol, 30% ethylene glycol and 40% PBS) until processing (*n* = 6 per group).

### Immunofluorescence

Free-floating sections were rinsed and blocked with 10% (w/v) normal donkey serum in Tris-buffered saline (TBS) for 1 h at room temperature. Then, the sections were incubated with primary antibodies for 48 h at 4°C. The following primary antibodies were used: mouse anti-Aβ monoclonal antibody (6E10; Covance, 1:500); mouse anti-phospho-tau (ser202, Thr205; AT-8; Thermo Fisher, 1:200); rabbit anti-ionized calcium-binding adapter molecule 1 (Iba1) antibody (Abcam, 1:200); and rabbit anti-glial fibrillary acidic protein (GFAP) antibody (Abcam, 1:200). The sections were then rinsed with TBS and incubated for 2 h at room temperature with the respective secondary antibodies, Alexa Fluor-555-conjugated donkey anti-mouse IgG (Jackson ImmunoResearch, 1:200), Alexa Fluor-488-conjugated donkey anti-mouse IgG (Jackson ImmunoResearch, 1:200) or Alexa Fluor-488-conjugated donkey anti-rabbit IgG (Jackson ImmunoResearch, 1:200). After a final rinse in TBS, the sections were mounted on chrome-alum gelatin-coated slides, air-dried and covered with glycerol (diluted in PBS, 1:1, v/v). Staining was visualized using fluorescence microscopy (Leica, Japan; *n* = 6 per group).

### Thioflavin-S Staining

Brain sections were incubated with a 0.5% thioflavin-S solution dissolved in distilled water containing 50% ethanol for 20 min and differentiated in 50% ethanol three times. Fluorescence imaging was performed using an Olympus fluorescence microscope (Olympus Corporation, Japan). To quantify the plaque load, the areas of 10 sections of the cortex and hippocampus in each group were calculated (*n* = 6 per group).

### Aβ ELISA

Aβ ELISA was performed as previously described (Gontier et al., 2015). In brief, the cortical and hippocampal tissues were thoroughly homogenized in TBS containing a protease inhibitor cocktail (Roche). The homogenates were then mixed 1:1 with 0.4% diethylamine (DEA), sonicated and centrifuged at 13,000 g for 30 min at 4°C. The supernatants were collected for the quantification of soluble Aβ. Meanwhile, the pellets were homogenized in 70% formic acid (FA). After sonication and centrifugation, the supernatants were collected for the analysis of insoluble Aβ. The Aβ concentrations were detected using human Aβ40 and Aβ42 ELISA kits (Invitrogen) following the manufacturer’s instructions. The normalized amounts of Aβ were expressed as pmol/mg of protein (*n* = 6 per group).

### BMP9 ELISA

The mice were sacrificed by decapitation, and the cortex and hippocampus were dissected on ice. Tissue samples were frozen immediately on dry ice and stored at −80°C until analysis. Tissues were homogenized in TBS containing a protease inhibitor cocktail (Roche). After centrifugation, the supernatants were collected. BMP9 levels were measured using a BMP9 ELISA kit according to the manufacturer’s protocol (R&D Biosciences). The absorbance at 450 nm was measured with a spectrophotometer. The normalized amounts of BMP9 were expressed as pg/100 mg of protein (*n* = 6 per group).

### Western Blot Analysis

Brain tissues were dissected and homogenized in RIPA buffer containing a cocktail of protease and phosphatase inhibitors, as previously described (Wang et al., 2016). Protein concentrations were determined using a bicinchoninic acid protein assay kit (Pierce). Equal amounts of total protein (20 μg) were electrophoresed on 10% SDS-PAGE gels, and then transferred onto nitrocellulose membranes (Bio-Rad). After the membranes were blocked with 5% BSA in TBST for 1 h at room temperature, the membranes were incubated with primary antibodies overnight at 4°C. The following primary antibodies were used: mouse anti-Aβ monoclonal antibody (6E10; Covance, 1:1000); rabbit anti-beta-secretase 1 (BACE1) antibody (Abcam, 1:1000); rabbit anti-insulin-degrading enzyme (IDE) antibody (Abcam, 1:1000); rabbit anti-neprilysin (NEP) antibody (Abcam, 1:5000); rabbit anti-receptor for advanced glycation endproducts (RAGE) antibody (Abcam, 1:2000); rabbit anti-low-density lipoprotein receptor-related protein 1 (LRP1) antibody (Abcam, 1:5000); rabbit anti-phospho-tau (Thr181; CST, 1:1000); rabbit anti-phospho-tau (Ser 396; CST, 1:1000); rabbit anti-phospho-tau (Ser 199; CST, 1:1000); rabbit anti-phospho-tau (Ser 356; CST, 1:1000); mouse anti-tau (Tau-5; Abcam, 1:1000); rabbit anti-phospho-GSK3β (Ser9; CST, 1:1000); rabbit anti-GSK3β (CST, 1:1000); rabbit anti-phospho-GSK3α (CST, 1:1000); rabbit anti-GSK3α (CST, 1:1000); rabbit anti-cyclin-dependent kinase 5 (CDK5; CST, 1:1000); rabbit anti-protein phosphatase 2 (PP2)A-C (CST, 1:1000); rabbit anti-phospho-PP2A-C (Y307; R&D Biosciences 1:1000), rabbit anti-demethylated PP2A-C (D-PP2A-C; CST, 1:1000), rabbit anti-PP2B (CST, 1:1000), rabbit anti-phospho-extracellular signal-regulated kinase 1 and 2 (ERK1/2; Abcam, 1:1000); rabbit anti-ERK1/2 (Abcam, 1:1000); rabbit anti-phospho-p38 mitogen-activated protein kinase (MAPK; Abcam, 1:1000); rabbit anti-p38 MAPK (Abcam, 1:1000); rabbit anti-phospho-Smad1/5/8 (CST, 1:1000); rabbit anti-Smad1/5/8 (CST, 1:1000); rabbit anti-Smad4 (Abcam, 1:5000); and mouse anti-GAPDH antibody (Abcam, 1:10,000). After being washed in TBST, the membranes were incubated with the respective IRDye 800CW-labeled or IRDye 680CW-labeled secondary antibodies (1:1000) for 1 h at room temperature. After a final wash in TBST, the signals were detected with an Odyssey Infrared Imaging System (LICOR Bioscience, Lincoln, NE). ImageJ (NIH) was utilized for protein band densitometry (*n* = 6 per group).

### Quantitative Real-time PCR

Total RNA was isolated from brain tissues using a total RNA kit (Omega) according to the manufacturer’s instructions. Reverse transcription was performed using a PrimeScript^TM^ II 1st Strand cDNA Synthesis kit (Takara), and the reaction mix was subjected to quantitative real-time PCR using a SYBR^®^ Premix Ex Taq^TM^ II (Tli RNaseH Plus) kit (Takara) to detect the corresponding mouse interleukin 1 beta (IL-1β), IL-6, tumor necrosis factor alpha (TNF-α), IL-4, IL-10, TGF-β, monocyte chemotactic protein 1 (MCP-1), and macrophage inflammatory protein 1 alpha (MIP-1α) transcription levels. A set of β-actin primers was used as an internal control for each specific gene amplification. The real-time value for each sample was compared using the comparative (CT) method, where the amount of target RNA (2^−ΔCT^) was normalized to the endogenous actin reference (ΔCT; Schmittgen and Livak, 2008; *n* = 6 per group). The primers used for quantitative real-time PCR are listed in Table 1.

**Table 1:** **Primers used for quantitative real-time PCR**.

Gene	Direction	Sequence
TNF-α	F	5′-CCCTCACACTCAGATCATCTTCT-3′
	R	5′-GCTACGACGTGGGCTACAG-3′
Il-1β	F	5′-TAGTCCTTCCTACCCCAATTTCC-3′
	R	5′-TTGGTCCTTAGCCACTCCTTC-3′
IL-6	F	5′-GCAACTGTTCCTGAACTCAACT-3′
	R	5′-ATCTTTTGGGGTCCGTCAACT-3′
IL-4	F	5′-GGTCTCAACCCCCAGCTAGT-3′
	R	5′-GCCGATGATCTCTCTCAAGTGAT-3′
IL-10	F	5′-TGGCCACACTTGAGAGCTGC-3′
	R	5′-TTCAGGGATGAAGCGGCTGG-3′
TGF-β	F	5′-CTCCCGTGGCTTCTAGTGC-3′
	R	5′-GCCTTAGTTTGGACAGGATCTG-3′
MIP-1α	F	5′-TTCTCTGTACCATGACACTCTGC-3′
	R	5′-CGTGGAATCTTCCGGCTGTAG-3′
MCP-1	F	5′-TAAAAACCTGGATCGGAACCAA-3′
	R	5′-GCATTAGCTTCAGATTTACGGGT-3′
β-Actin	F	5′-GGCTGTATTCCCCTCCATCG-3′
	R	5′-CCAGTTGGTAACAATGCCATGT-3′

### Statistical Analysis

All reported experiments were performed in triplicate and conducted at least three times. Data are reported as the mean ± standard deviation (SD). Statistical analyses were performed using GraphPad Prism software (version 5, GraphPad Software). Student’s *t* test was performed for statistical analyses between two groups. ANOVA followed by Bonferroni’s *post hoc* test was used when three or more groups were compared. Statistical significance was defined as *p* < 0.05.

## Results

### BMP9 Was Successfully Delivered into the Brain via the Intranasal Pathway

**Figure 1:**
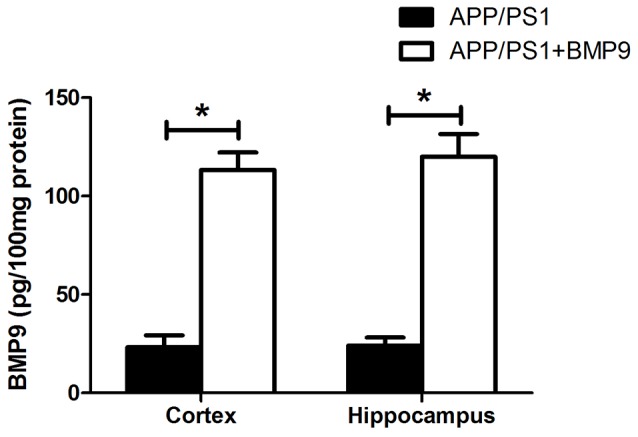
**Bone morphogenetic protein 9 (BMP9) was successfully delivered into the brain via the intranasal pathway.** BMP9 concentrations in the cortex and hippocampus were measured using a BMP9 ELISA kit. Values are represented as the mean ± SD. ANOVA followed by Bonferroni’s *post hoc* test. *p < 0.05, *n* = 12.

Given that BMP9 is unlikely to cross the blood-brain barrier (BBB), the intranasal pathway was used in the present study to investigate the therapeutic effects of BMP9 in a transgenic model of AD. We first measured the concentration of BMP9 in the cortex and hippocampus using a BMP9 ELISA kit. As shown in Figure 1, the level of BMP9 was significantly increased in the brains of BMP9-treated APP/PS1 mice (cortex, 113.5 ± 12.5 pg/100 mg; hippocampus, 121.0 ± 16.3 pg/100 mg) compared with the vehicle-treated group (cortex, 23.3 ± 7.8 pg/100 mg; hippocampus, 24.5 ± 5.9 pg/100 mg; ANOVA, *p* < 0.05, *n* = 12), indicating that BMP9 was successfully delivered into the brain via the intranasal pathway.

### BMP9 Improved Cognitive Function in APP/PS1 Mice

**Figure 2:**
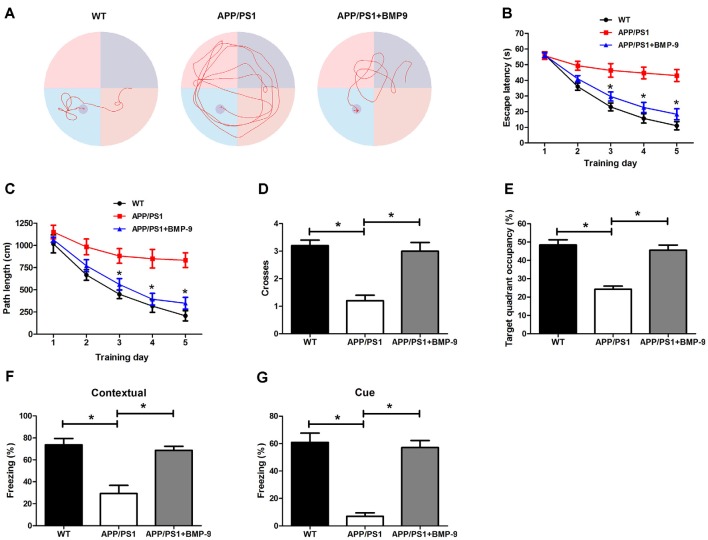
**BMP9 treatment rescued impaired spatial and associative learning and memory in APP/PS1 mice. (A)** Typical swimming traces during the acquisition test showing the effects of BMP9 on the spatial memory of APP/PS1 mice. **(B)** Escape latency in the Morris water maze plotted against the training days. **(C)** Average distance to find the platform. **(D)** Times crossing the target sites after retrieval of the platform. **(E)** Percentage of time spent in the target quadrant during a 60-sec probe trial. **(F,G)** Mean freezing behavior in the contextual** (F)** and cued **(G)** fear conditioning tests. Values are represented as the mean ± SD. ANOVA followed by Bonferroni’s *post hoc* test. *p < 0.05, *n* = 12.

To investigate whether BMP9 could ameliorate AD-associated learning and memory deficits, cognitive function was first tested using the Morris water maze. During acquisition, WT mice showed a day-to-day decrease in escape latency, whereas APP/PS1 mice displayed a significantly longer escape latency (Figures 2A,B, ANOVA, *p* < 0.05, *n* = 12). In addition, the path length to find the platform was longer for APP/PS1 mice than for WT mice (Figure 2C, ANOVA, *p* < 0.05, *n* = 12). During the probe trial test, APP/PS1 mice crossed the platform less often and spent less time within the target quadrant than did WT mice (Figures 2D,E, ANOVA, *p* < 0.05, *n* = 12). Strikingly, the impaired spatial learning and memory exhibited by APP/PS1 mice were rescued by treatment with BMP9, as demonstrated by the BMP9-treated APP/PS1 mice showing a reduced escape latency, shortened path length to the platform, increased platform crossing, and increased target quadrant occupation, which were all comparable to those displayed by WT mice (Figures 2B–E, ANOVA, *p* < 0.05, *n* = 12).

We next investigated the effects of BMP9 on APP/PS1 mouse associative learning and memory using a standard conditioned fear memory paradigm (Fu et al., 2016). For both contextual and cued fear conditioning, APP/PS1 mice exhibited decreased freezing behavior than the WT mice, indicating that the APP/PS1 mice had an impaired associative memory (Figures 2F,G, ANOVA, *p* < 0.05, *n* = 12). However, the intranasal BMP9 treatment restored the impaired associative memory of APP/PS1 mice, as indicated by augmented freezing responses in both the contextual and cued fear conditioning tests (Figures 2F,G, ANOVA, *p* < 0.05, *n* = 12). These data indicate that BMP9 can improve the cognitive function of APP/PS1 mice.

### BMP9 Reduced Aβ Deposition and Levels in APP/PS1 Mice

**Figure 3:**
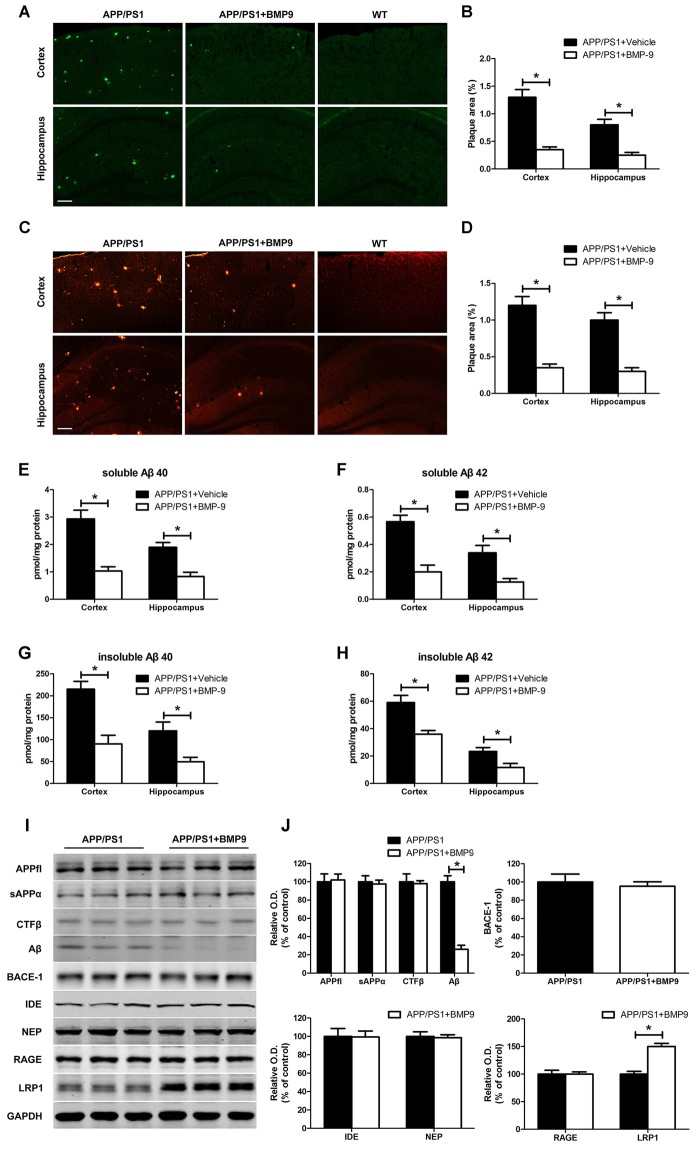
**BMP9 treatment diminished amyloid plaque pathology and Aβ levels in APP/PS1 mice. (A,B)** Brain sections of the cortex and hippocampus were stained with thioflavin-S, and the proportions of Aβ-positive area were calculated. Bar = 100 μm. **(C,D)** The Aβ plaques in the cortex and hippocampus were estimated after immunofluorescence staining with Aβ antibodies (6E10), and the proportions of positive area were calculated. Bar = 100 μm. **(E–H)** The levels of soluble and insoluble Aβ were measured using ELISA. **(I)** Representative western blots of APPfl, sAPPα, C-terminal fragment beta (CTFβ), Aβ, BACE1, IDE, NEP, RAGE, lipoprotein receptor-related protein 1 (LRP1), and GAPDH in the hippocampus homogenates from APP/PS1 mice treated with vehicle or BMP9. **(J)** Densitometric analyses of the immunoreactivities to the antibodies shown in the previous panel. Values are represented as the mean ± SD. Student’s *t* test or ANOVA followed by Bonferroni’s *post hoc* test. *p < 0.05, *n* = 6.

To investigate the effects of BMP9 on Aβ deposition, we performed thioflavin-S staining for mature amyloid plaques and Aβ immunostaining (6E10) for total amyloid plaques. As shown in Figures 3A,B, BMP9-treated APP/PS1 mice exhibited lower areas of thioflavin-S-stained plaques than did vehicle-treated mice (ANOVA, *p* < 0.05, *n* = 6). Immunofluorescence Aβ brain deposition studies confirmed this observation, showing that the Aβ immunopositive areas occupied by 6E10 immunoreactions were significantly lower in BMP9-treated APP/PS1 mice than in vehicle-treated mice (Figures 3C,D, ANOVA, *p* < 0.05, *n* = 6). To extend on the amyloid plaque results, the levels of different Aβ isoforms in the cortex and hippocampus were measured by ELISA. BMP9-treated APP/PS1 mice showed a marked decrease in the brain levels of both soluble (DEA extractable) and insoluble (FA extractable) Aβ40 and Aβ42 compared with vehicle-treated mice (Figures 3E–H, ANOVA, *p* < 0.05, *n* = 6).

To investigate the potential mechanisms responsible for the effect of BMP9 on Aβ, we examined the metabolism of its precursor, the Aβ precursor protein (APP), using western blot analyses. Consistent with the immunofluorescence analysis, the level of Aβ in the hippocampus of BMP9-treated APP/PS1 mice was significantly lower than that in vehicle-treated mice. However, the levels of full-length APP (APPfl), secreted APP alpha (sAPPα), C-terminal fragment beta (CTFβ), and BACE1 remained unchanged (Figures 3I,J, ANOVA, *p* > 0.05, *n* = 6). We also analyzed two of the major proteases involved in Aβ degradation, IDE and NEP, and found that the levels of these proteins did not differ between the BMP9-treated and vehicle-treated groups (Figures 3I,J, ANOVA, *p* > 0.05, *n* = 6). We further analyzed the two transporters mediating the transportation of Aβ, RAGE and LRP1, and observed that the level of LRP1, which mediates the efflux of Aβ, was significantly increased in BMP9-treated APP/PS1 mice compared with vehicle-treated mice (Figures 3I,J, ANOVA, *p* < 0.05, *n* = 6). In contrast, the level of RAGE, which is involved in the influx of Aβ, remained unchanged (Figures 3I,J). These results suggest that BMP9 attenuates the Aβ burden in the brains of APP/PS1 mice, probably by promoting the LRP1-mediated efflux of Aβ.

### BMP9 Inhibited Tau Hyperphosphorylation in APP/PS1 Mice

**Figure 4:**
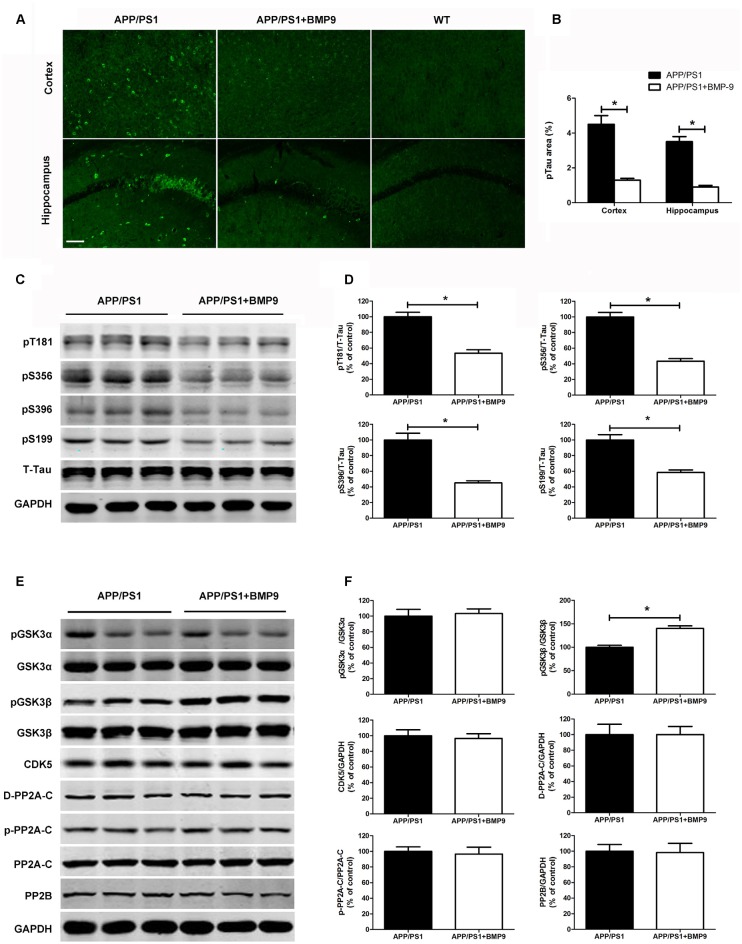
**BMP9 treatment attenuated tau phosphorylation in APP/PS1 mice. (A)** Brain sections of the cortex and hippocampus were stained with anti-phospho-tau (ser202, Thr205; AT-8). **(B)** The proportions of AT-8-positive area were calculated. Bar = 100 μm. **(C)** Representative western blots of phosphorylated tau at residues T181, S356, S396, and S199, as well as those of total tau and GAPDH in the hippocampus homogenates from APP/PS1 mice treated with vehicle or BMP9. **(D)** Densitometric analyses of the immunoreactivities to the antibodies shown in the previous panel. **(E)** Representative western blots of pGSK3α, GSK3α, pGSK3β, GSK3β, cyclin-dependent kinase 5 (CDK5), PP2A-C, pPP2A-C (Y307), demethylated PP2A-C (D-PP2A-C), PP2A-B and GAPDH in the hippocampus homogenates from APP/PS1 mice treated with vehicle or BMP9. **(F)** Densitometric analyses of the immunoreactivities to the antibodies shown in the previous panel. Values are represented as the mean ± SD. Student’s *t* test or ANOVA followed by Bonferroni’s *post hoc* test. *p < 0.05, *n* = 6.

Tangles composed of hyperphosphorylated tau are another important pathological characteristic of AD. To investigate whether BMP9 could affect tau pathology, coronal brain sections of the cortex and hippocampus were stained with antibodies against phospho-tau (Ser202, Thr205; AT-8). As shown in Figures 4A,B, the proportions of areas with AT-8-positive neurons in the cortex and hippocampus were significantly lower in BMP9-treated APP/PS1 mice than in vehicle-treated mice (ANOVA, *p* < 0.05, *n* = 6). Consistently, the western blot analysis further showed that tau phosphorylation at multiple sites, including Ser396, Ser199, Ser356 and Thr181, was significantly diminished in the brains of BMP9-treated APP/PS1 mice (Figures 4C,D, ANOVA, *p* < 0.05, *n* = 6).

To study the mechanisms underlying the effects of BMP9 on tau phosphorylation, we next examined kinases and phosphatases that are considered major regulators of its posttranslational modifications (Dolan and Johnson, 2010). As shown in Figures 4E,F, compared with vehicle-treated mice, the brains of BMP9-treated mice showed a significantly increased level of phosphorylated GSK3β (Ser9; Student’s* t* test, *p* < 0.05, *n* = 6). In contrast, no differences were found among the levels of total or phosphorylated GSK3α, CDK5, PP2A-C, pPP2A-C (Y307), D-PP2A-C, or PP2B (Student’s *t* test, *p* > 0.05, *n* = 6). Since GSK3β phosphorylation at Ser9 leads to the inhibition of its activity, our findings suggest that BMP9 administration suppresses tau hyperphosphorylation, probably by deactivating GSK3β.

### BMP9 Attenuated Neuroinflammation in APP/PS1 Mice

**Figure 5:**
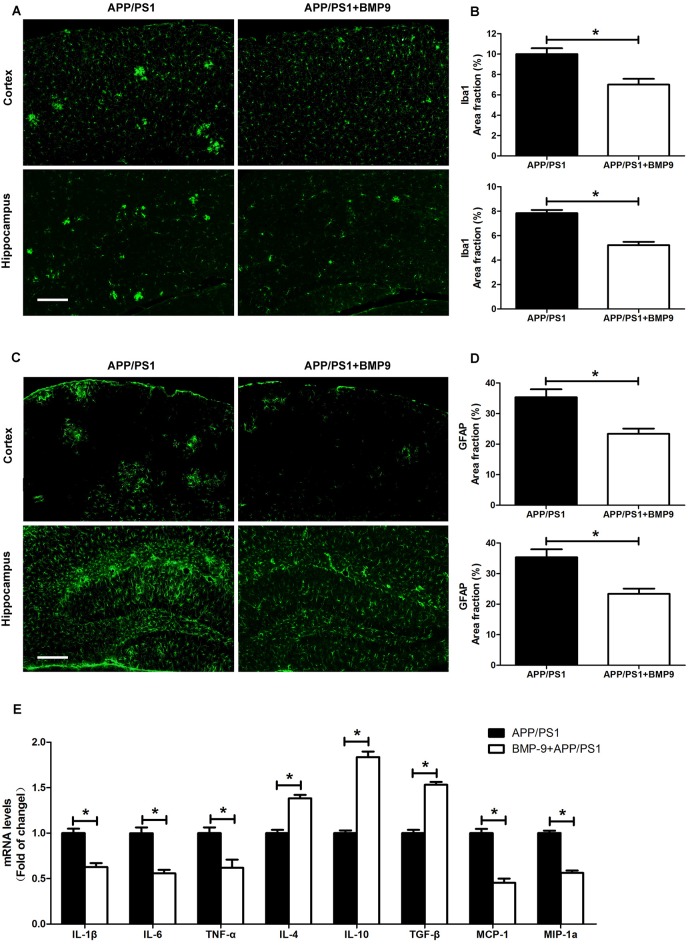
**BMP9 treatment reduced neuroinflammation in APP/PS1 mice. (A)** Brain sections of the cortex and hippocampus were stained with anti-Iba1. Bar = 100 μm. **(B)** The proportions of Iba1-positive area were calculated. **(C)** Brain sections of the cortex and hippocampus were stained with anti-glial fibrillary acidic protein (GFAP). Bar = 100 μm. **(D)** The proportions of GFAP-positive area were calculated. **(E)** The transcripts of cytokines and chemokines in the brains of APP/PS1 mice treated with vehicle or BMP9 were measured by quantitative RT-PCR. Values are represented as the mean ± SD. Student’s *t* test or ANOVA followed by Bonferroni’s *post hoc* test. *p < 0.05, *n* = 6.

Neuroinflammation characterized by the activation of microglia and astrocytes is an important component of AD (Heneka et al., 2015). As such, we examined whether intranasal BMP9 administration could inhibit glial activation in APP/PS1 mice. Coronal sections of the cortex and hippocampus were stained with an antibody against Iba-1, which is indicative of pronounced microglia activation. We found that BMP9-treated APP/PS1 mice displayed less intense immunohistochemical Iba-1 labeling in the cortex and hippocampus than did vehicle-treated mice (Figures 5A,B, Student’s *t* test, *p* < 0.05, *n* = 6), suggesting that BMP9 exerted an inhibitive effect on microglial activation. Similar to microglia, astrocytes are also strongly activated during the progression of AD and closely associated with disease pathology (Birch, 2014). Compared with the vehicle-treated mice, BMP9-treated APP/PS1 mice exhibited a 30–40% decrease in the proportions of GFAP-immunoreactive areas in the cortex and hippocampus (Figures 5C,D, Student’s *t* test, *p* < 0.05, *n* = 6), indicating that BMP9 also suppresses astrocyte activation. Activated microglia and astrocytes secrete inflammatory cytokines and chemokines, causing neuronal and synaptic dysfunction. We found that the transcription levels of proinflammatory cytokines (IL-1β, IL-6 and TNF-α) and chemokines (MCP-1 and MIP-1α) were significantly decreased in BMP9-treated APP/PS1 mice (Figure 5E, ANOVA, *p* < 0.05, *n* = 6). In contrast, the transcripts of anti-inflammatory cytokines (IL-4, IL-10 and TGF-β) were profoundly increased in BMP9-treated APP/PS1 mice (Figure 5E, ANOVA, *p* < 0.05, *n* = 6). Together, these results indicate that intranasal BMP9 administration attenuates neuroinflammation in APP/PS1 mice.

### BMP9 Activated Smad-Dependent Signaling in APP/PS1 Mice

**Figure 6:**
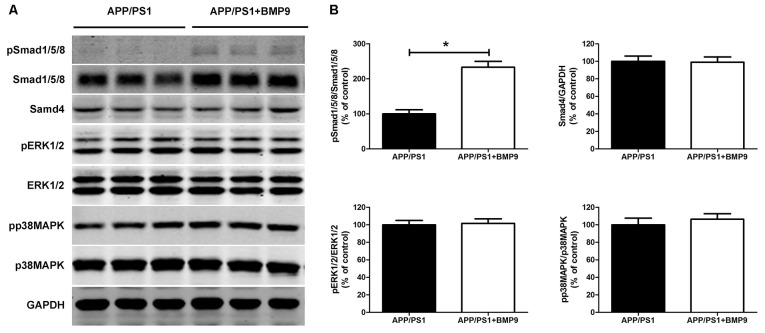
**BMP9 acted via a canonical Smad-dependent signaling pathway in APP/PS1 mice. (A)** Representative western blots of pSmad1/5/8, Smad1/5/8, Smad4, pERK1/2, ERK1/2, pp38 mitogen-activated protein kinase (MAPK), p38 MAPK and GAPDH in the hippocampus homogenates from APP/PS1 mice treated with vehicle or BMP9. **(B)** Densitometric analyses of the immunoreactivities to the antibodies shown in the previous panel. Values are represented as the mean ± SD. Student’s *t* test. *p < 0.05, *n* = 6.

BMP9, acting on a tetrameric receptor complex, transduces signals mainly via Smad-dependent signaling pathways (Wu et al., 2016). Recently, crosstalk has also been found between Smad and MAPK signaling pathways. To investigate which pathway mediated the beneficial effects of BMP9 in APP/PS1 mice, the protein levels of pSmad1/5/8, Smad1/5/8, Smad4, pERK1/2, ERK1/2, pp38MAPK and p38MAPK in the hippocampal homogenates were detected by western blot. As shown in Figure 6, intranasal BMP9 administration significantly increased the levels of pSmad1/5/8 (Student’s *t* test, *p* < 0.05, *n* = 6), while having no effects on the phosphorylation of ERK1/2 or p38 MAPK (Student’s *t* test, *p* > 0.05, *n* = 6). These data suggest that BMP9 exerts beneficial effects against AD-like pathological and memory deficits, likely via canonical Smad-dependent signaling pathways.

## Discussion

In the present study, we found that the intranasal administration of BMP9 significantly reduced the amyloid plaque load and Aβ levels in the brains of APP/PS1 mice, probably by promoting the efflux of Aβ mediated by LRP1. In addition, BMP9 inhibited tau hyperphosphorylation in the cortex and hippocampus, possibly by the deactivation of GSK3β. Further, BMP9 attenuated the activation of astrocytes and microglia. More importantly, BMP9 rescued the impaired spatial and associative learning and memory of APP/PS1 mice. Together, our results suggest that BMP9 ameliorates cognitive impairments in AD by targeting multiple key pathways in the disease pathogenesis.

The BBB prevents foreign materials, particularly large and charged materials, from entering the brain. During past decades, the intranasal pathway has been developed as a novel and non-invasive method for delivering therapeutic substances directly to the central nervous system (Chapman et al., 2013). Various studies have confirmed the safety of a variety of intranasal compounds, including insulin, leptin, oxytocin, insulin-like growth factor 1 and even stem cells (De Rosa et al., 2005; Benedict et al., 2007; Chapman et al., 2013). Nonetheless, the possible adverse effects of intranasal drugs on the local nasal mucosa should not be neglected. Given that BMP9 is unlikely to cross the BBB, the intranasal pathway was used in the present study to investigate the therapeutic effects of BMP9 in a transgenic mouse model of AD. We found a significantly increased level of BMP9 in both the cortex and hippocampus of BMP9-treated APP/PS1 mice compared with vehicle-treated APP/PS1 mice, indicating that BMP9 was successfully delivered to the brain. Strikingly, we further found that the impaired spatial memory function assessed by the Morris water maze was significantly reversed by a 4-week intranasal BMP9 treatment. Consistently, the associative memory function evaluated by the fear conditioning test in APP/PS1 mice was also profoundly ameliorated by BMP9. To the best of our knowledge, we are the first to show that intranasal BMP9 administration can ameliorate cognitive impairments in AD transgenic mice.

In line with the behavioral studies, we observed that the brains of BMP9-treated APP/PS1 mice had significantly less Aβ deposition than did vehicle-treated mice. This observation is consistent with a previous report showing that a 7-day intracerebroventricular infusion of BMP9 reduced the number of Aβ42-positive amyloid plaques in the hippocampus and cerebral cortex of 5- and 10-month-old APP.PS1/CHGFP mice (Burke et al., 2013). The decreased Aβ plaque burden was further confirmed by the findings that both the soluble and insoluble Aβ1–40 and Aβ1–42 fractions were much lower in the brains of BMP9-treated APP/PS1 mice. In contrast to our observations, Burke et al. (2013) found no significant effects of BMP9 on soluble Aβ40 and Aβ42 levels. This discrepancy may be partially derived from the different BMP9 treatment periods. The administration of BMP9 lasted for 4 weeks in our study, while it only lasted 7 days in the study by Burke et al. (2013). BMP9 was suggested to slow the aggregation and deposition of Aβ in a rapid manner; however, the clearance of Aβ from the brain was much slower (Mellott et al., 2014).

To unravel the possible mechanisms underlying the beneficial effect of BMP9 on Aβ plaques and burden, the APP proteolytic, Aβ catalytic, and Aβ transportation pathways were investigated. The protein levels of total APP, sAPPα, CTFβ and BACE remained unchanged, indicating that BMP9 does not affect APP processing. Meanwhile, BMP9 did not affect the catalysis of Aβ, as demonstrated by the similar protein levels of IDE and NEP, which are involved in the degradation of Aβ. Strikingly, compared to vehicle-treated APP/PS1 mice, an increased LRP1 protein level was found in the brains of BMP9-treated mice, while the level of RAGE, which mediates the influx of Aβ, was unaffected. LRP1 is ubiquitously expressed in various cells in the brain, including neurons, glial cells, and brain endothelial cells. The conditional knockout of LRP1 in the neurons of APP/PS1 mice resulted in exacerbated amyloid pathology in the brain (Kanekiyo et al., 2013). Studies have also shown that LRP1 in astrocytes and microglia controlled the uptake of extracellular Aβ and further mediated glial activation (LaDu et al., 2000; Laporte et al., 2004). Brain endothelial cell-specific LRP1 deletion in AD transgenic mice led to elevated levels of soluble brain Aβ and amyloid plaques, resulting in aggravated cognitive deficits (Kanekiyo et al., 2012; Storck et al., 2016). These data suggest that LRP1 in the brain plays a critical role in the clearance of Aβ. Therefore, the significantly increased level of LRP1 in BMP9-treated APP/PS1 mice suggests that BMP9 reduces Aβ accumulation and plaque burden, probably by promoting the LRP1-mediated efflux of Aβ. Further studies using LRP1 knockout APP/PS1 mice are warranted to thoroughly clarify the role of LRP1 in the BMP9-induced clearance of Aβ.

The intracellular aggregation of hyperphosphorylated tau in the form of NFTs is another pathological hallmark of AD (Ballatore et al., 2007; Small and Duff, 2008). Hyperphosphorylated tau detaches from microtubules and accumulates in paired helical filaments, leading to axonal dysfunction and synaptic failure (Stoothoff and Johnson, 2005; Spillantini and Goedert, 2013). Furthermore, tau hyperphosphorylation is more closely correlated with the degree of dementia in AD than are amyloid plaques (Braak and Braak, 1991; Ballatore et al., 2007). Significant cognitive impairment was consistently found in different transgenic mouse models of tauopathy (Polydoro et al., 2009; Sydow et al., 2011; Van der Jeugd et al., 2012). These observations led to the hypothesis that tau pathology could be a novel target for AD treatment (Himmelstein et al., 2012). In the present study, compared to vehicle-treated mice, BMP9-treated APP/PS1 mice had a significantly decreased brain level of hyperphosphorylated tau, as demonstrated by the decreased immunofluorescence intensity of AT8 in both the cortex and hippocampus. These observations were further confirmed by the findings that intranasal BMP9 significantly suppressed the hyperphosphorylation of tau at several sites, including Ser396, Ser199, Ser356, and Thr181. These data strongly indicate that BMP9 could also repress the hyperphosphorylation of tau in APP/PS1 mice.

The phosphorylation status of tau is controlled by the activities of various kinases and phosphatases. Kinases involved in the phosphorylation of tau include GSK3β, CDK5, Fyn, and microtubule affinity-regulating kinases. Of these, GSK3β and CDK5 are considered the main tau kinases responsible for phosphorylating the majority of epitopes present in paired helical filament tau. In addition, tau is dephosphorylated mainly by PP2A and to a lesser extent by PP2B, PP1, and PP5 (Planel et al., 2001; Dolan and Johnson, 2010). Therefore, the present study focused on the activated forms of GSK3β, CDK5, and PP2A. We found that BMP9 selectively increased the phosphorylation of GSK3β at Ser9, while having no effects on CDK5 or PP2A. In accordance with our observations, BMP9 has also been reported to suppress the activity of GSK3β in preadipocytes (Liu et al., 2014). GSK3β, initially identified as a kinase involved in glycogen metabolism, is now considered the major physiological and pathological tau kinase (Rayasam et al., 2009; Hernandez et al., 2013). GSK3β activation has induced tauopathy-related phenotypes in mouse models (Terwel et al., 2008; Crespo-Biel et al., 2014), and GSK3β inhibitors have exhibited beneficial effects against tauopathy and neurodegeneration in tau transgenic mice (Uno et al., 2009; Martinez et al., 2011). These findings suggest that GSK3β inhibitors may have therapeutic potential in the treatment of AD. Given that BMP9 significantly upregulated the phosphorylation of pGSK3β at Ser9, which led to its inactivation, we posit that BMP9 suppresses tau hyperphosphorylation in the brains of APP/PS1 mice by inhibiting the activity of GSK3β. Studies using specific GSK3β activators are needed to further confirm the role of GSK3β in the inhibitive effect of BMP9 on tau hyperphosphorylation.

Neuroinflammation, manifested by the activation of microglia and astrocytes, is another important component of AD pathology and contributes to disease progression (Heneka et al., 2015; Calsolaro and Edison, 2016). In AD, microglia have been implicated in the generation of chronic neurotoxic inflammation around amyloid plaques, while being unable to clear the plaques effectively (Pimplikar, 2014; Pasqualetti et al., 2015). In addition, activated microglia secrete a wide range of cytokines and other proinflammatory factors, which contributes to neurodegeneration and exacerbate inflammatory reactions by recruiting and stimulating astrocytes (Rubio-Perez and Morillas-Ruiz, 2012; Ransohoff, 2016). More importantly, non-steroidal anti-inflammatory drugs (NSAIDs) dramatically reduced the risk for AD, delayed disease onset, ameliorated symptomatic severity, and slowed cognitive decline (In ’t Veld et al., 1998; Arvanitakis et al., 2008; Breitner et al., 2009; Lehrer, 2014). Thus, targeting neuroinflammation has been proposed as a novel AD treatment strategy (Pimplikar, 2014; Bronzuoli et al., 2016). Noticeably, we showed here that intranasal BMP9 administration significantly suppressed the activation of microglia and astrocytes. In contrast with our results, Burke et al. (2013) demonstrated that BMP9 did not affect hippocampal gliosis in APP.PS1/CHGP mice. The reasons leading to this discrepancy are complex. First, the BMP9 treatment period was longer in our study. Second, astrocyte activation was not evaluated by immunofluorescence in Burke’s study. Third, the state of microglial activation was not assessed in their study. In line with our immunofluorescence findings, we further demonstrated that BMP9 significantly decreased the levels of proinflammatory cytokines (IL-1β, IL-6, TNF-α) and chemokines (MCP-1, MIP-1α), while increasing the levels of anti-inflammatory cytokines (IL-4, IL-10, TGF-β). This switch from proinflammatory to anti-inflammatory cytokine secretion supports the notion that BMP9 could suppress neuroinflammation in APP/PS1 mice. Together, our observations indicate that intranasally administered BMP9 also acts as an efficient inhibitor of neuroinflammation in APP/PS1 mice.

Like other members of the TGF-β superfamily, BMP9 functions via the Smad1/5/8 signaling pathway (Bandyopadhyay et al., 2013; Zhong and Zou, 2014). The activation of this Smad signaling pathway was necessary not only for BMP9-mediated osteogenic stem cell differentiation (Wang et al., 2013; Chen et al., 2016) but was also essential for BMP9-induced human endothelial cell quiescence (Rostama et al., 2015). Furthermore, the Smad signaling pathway may be responsible for the induction of the cholinergic phenotype by BMP9 in the basal forebrain (López-Coviella et al., 2006). In agreement with these studies, we demonstrated in the present study that intranasal BMP9 administration significantly promoted the phosphorylation of Smad1/5/8 in the hippocampus of APP/PS1 mice. Meanwhile, the MAPK signaling pathways, which are also suggested to be involved in the beneficial effects of BMP9, were unaffected, as demonstrated by the unchanged pERK and pp38MAPK levels. These results indicate that BMP9 exerts a beneficial effect against cognitive impairment via a Smad-dependent signaling pathway in APP/PS1 mice.

Increasing evidence shows that there is an interaction between BMP9 and the Wnt/β-catenin signaling pathway (Liu et al., 2014). We should bear in mind that activation of the canonical Wnt pathway would lead to the phosphorylation and subsequent deactivation of GSK3β (Wu and Pan, 2010). Interestingly, we found in the present study that BMP9 significantly increased the levels of phosphorylated GSK3β in APP/PS1 mice. We hypothesize that crosstalk between the Smad and Wnt/β-catenin signaling pathways may be responsible for the deactivation of GSK3β by BMP9 in APP/PS1 mice. Further studies using specific protein kinase inhibitors or siRNAs are warranted to verify this hypothesis.

In conclusion, we uncovered an application of BMP9 for the treatment of AD in a transgenic model by targeting multiple key pathways, including Aβ clearance mediated by LRP1, tau hyperphosphorylation mediated by GSK3β, and neuroinflammation. These data suggest that BMP9 holds potential as a promising candidate for the treatment of AD. Clinical trials are warranted to verify the efficacy of BMP9 in AD patients.

## Author Contributions

ZW and HZ designed the research work; ZW and WW performed the intranasal delivery of BMP9; ZW, WW and LX performed the behavioral analyses, histopathological analyses, and biochemical analyses. ZW and XB performed the RT-PCR studies. ZW and HZ interpreted the data and drafted the manuscript. All authors read and approved the final manuscript.

## Conflict of Interest Statement

The authors declare that the research was conducted in the absence of any commercial or financial relationships that could be construed as a potential conflict of interest.
